# Delayed Diagnosis of Locally Acquired Lyme Disease, Central North Carolina, USA 

**DOI:** 10.3201/eid3003.231302

**Published:** 2024-03

**Authors:** Ross M. Boyce, Peyton Pretsch, Kay Tyrlik, Abigail Schulz, Dana A. Giandomenico, Alexis M. Barbarin, Carl Williams

**Affiliations:** University of North Carolina at Chapel Hill, Chapel Hill, North Carolina, USA (R.M. Boyce, P. Pretsch, K. Tyrlik, D.A. Giandomenico);; University of Illinois College of Medicine at Peoria, Peoria, Illinois, USA (A. Schulz);; North Carolina Department of Health and Human Services, Raleigh, North Carolina, USA (A.M. Barbarin, C. Williams)

**Keywords:** Lyme disease, ticks, tickborne disease, vector-borne diseases, zoonoses, North Carolina, bacteria, United States

## Abstract

Healthcare providers in North Carolina, USA, have limited experience diagnosing and managing Lyme disease because few cases occur annually statewide. We outline the prolonged diagnostic course for a patient with locally acquired Lyme disease in North Carolina. This case highlights the need for greater awareness and professional education.

Historically, the burden of Lyme disease has been concentrated in the Northeast and upper Midwestern regions of the United States (*1*). Recent data suggest a southward expansion into areas of southwestern Virginia and western North Carolina (*2*,*3*). Although North Carolina frequently reports some of the highest incidence rates of spotted fever rickettsiosis and ehrlichiosis (*4*), Lyme disease transmission has been less intense than in neighboring states to the north (*5*). Black-legged ticks (*Ixodes scapularis*) have long been found in North Carolina, and speculation exists that the lower Lyme disease incidence may be attributable to differences in blood-meal seeking behaviors between the northern- and southern-origin ticks (*6*,*7*). Although North Carolina has seen an increase in cases, many clinicians have limited experience with Lyme disease, and diagnostic errors are common (*8*,*9*). We describe a case of Lyme disease diagnosed in an otherwise healthy woman living in central North Carolina who had no history of travel. 

## The Case

In mid-July, a generally healthy woman in her late 60s went biking around her neighborhood in the suburbs north of Raleigh, North Carolina. After the ride, she felt dehydrated, lightheaded, and excessively fatigued for the level of exertion. Four days later, she noted a large erythematous rash on the right side of her neck (Figure). She also had a fever reaching 38.6°C. Results of an antigen-based COVID-19 rapid test were negative. She treated her symptoms with acetaminophen.

**Figure:**
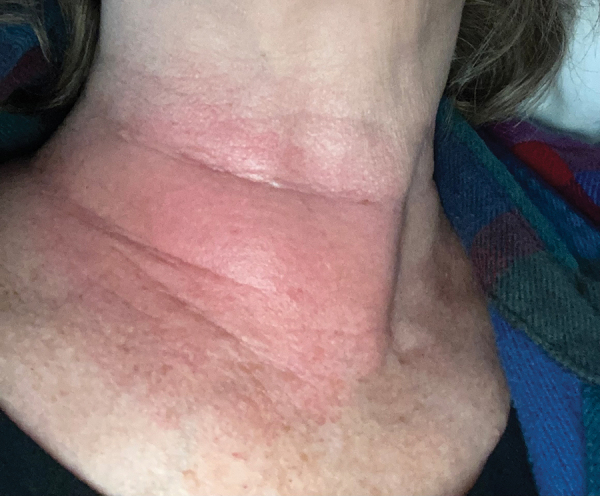
Erythematous rash on the right side of the neck of a patient with Lyme disease, central North Carolina, USA.

Approximately 5 days after the rash appeared, she went to her primary care physician (PCP) for her annual physical (Table). By that time, the fever had resolved, but the rash was still present. Additional symptoms included a severe frontal headache and bilateral ear pain. Her PCP diagnosed her with cellulitis and prescribed a 10-day course of cephalexin. After starting antibiotics, the patient felt subjectively better. However, the headache returned 2 days later. She contacted her PCP, who changed her antibiotic to double-strength trimethoprim/sulfamethoxazole out of concern that the headache was a side effect of cephalexin.

**Table:** Select clinical and laboratory information for a patient with Lyme disease, central North Carolina, USA*

Characteristic	Range	Day of illness (provider)				
		Day 10 (PCP)	Day 28 (PCP)		Day 43 (PCP)	Day 83 (ID clinic)
Signs/symptoms						
Fever >38.0°C		X				
Rash		X				
Fatigue		X				
Headache				X		
Itching				X		
Left-sided ear pain				X	X	
Left sided asymmetric smile					X	
Inability to close left eye					X	
Cognitive impairment					X	X
Other						
Laboratory testing						
Complete blood count						
Leukocytes, cells/mm^3^	4.0–11.0			6.1		
Hemoglobin, mg/dL	12.9–16.0			13.3		
Platelets, × 10^9^/L	150–400			193		
Metabolic panel						
Sodium, mmol/L	136–145			138		
Potassium, mmol/L	3.5–5.5			4.0		
Creatinine, mg/dL	0.6–1.30			0.70		
Liver function, U/L						
Alkaline phosphatase	38–126					
Aspartate aminotransferase	0–39					
Alanine aminotransferase	0–52					
C-reactive protein, mg/L	<10			10.2		<4.0
Sedimentation rate, mm/h	0–30			42		16
Tick-borne disease testing						
Lyme EIA	<0.91					Positive
Lyme Western blot						
IgM	0 of 3 bands					p41, p39, p23
IgG	0 of 10 bands					p66, p45, p41, p39, p23, p18
SFGR IgG	<1:64					<1:64
*Ehrlichia* IgG	<1:64					<1:64
α-gal IgE, kUA/L	<0.35†					0.43
Other infectious diseases						
HIV antigen/antibody	Nonreactive					Nonreactive
Syphilis antibody RPR	Nonreactive					Nonreactive
Diagnosis		Cellulitis			Bell’s palsy	Lyme disease
Treatment		Cephalexin, bactrim		Erythromycin	Valacyclovir, prednisone	Doxycycline

*EIA, enzyme immunoassay; ID, infectious disease; PCP, primary care provider; RPR, rapid plasma reagin; SFGR, spotted fever group *Rickettsia*; X, present. †Reference range <0.1 kUA/L (kUA/L level of allergy): <0.35 absent, 0.35–0.69 low, 0.70–3.49 moderate, 3.50–17.5 high, >17.5 very high.

The headaches persisted after the antibiotic change, and the next day the patient visited a local emergency department. Results of basic laboratory evaluations, including a complete blood count and comprehensive metabolic panel, were unremarkable. She underwent a noncontrast computed tomography scan of the head, which was interpreted as without findings that would explain her symptoms. She was subsequently discharged to home.

Ten days later, the patient returned to her PCP for follow-up and was seen by the on-call provider. She still reported pain in her ears and that the pain in the left ear was more severe than the right. She was now experiencing diffuse pruritis, which was thought to be caused by trimethoprim/sulfamethoxazole. The antibiotic was discontinued because the rash appeared to be resolving. However, she also noted more dyspnea with exertion. Additional laboratory testing was ordered, including a complete blood count, comprehensive metabolic panel, C-reactive protein, and erythrocyte sedimentation rate; the erythrocyte sedimentation rate was slightly elevated (Table). The patient was prescribed erythromycin drops for otitis media. A referral to cardiology was placed for evaluation of the exertional dyspnea.

After that visit, the patient became increasingly forgetful, withdrawn, and unable to perform basic cognitive tasks (e.g., simple calculations), which was noticed by her adult children. Two weeks later, ≈1 month after the rash began, she had onset of a left-sided facial droop. On evaluation, her PCP noted that she was unable to close her left eye and her smile was asymmetric on the same side. She was diagnosed with Bell’s palsy and prescribed a 1-week course of prednisone and valacyclovir. The facial nerve symptoms slowly improved and eventually resolved over the next week.

The next month, the patient reported more back pain with spasms that radiated into the cervical spine and neck. She underwent magnetic resonance imaging of the spine, which demonstrated degenerative changes but no findings that would explain her symptoms. Her children remained concerned about her cognitive status, anorexia, and unintentional 10-pound weight loss, and they requested additional consultations, including with a subspecialist in infectious diseases.

The patient was seen in an outpatient infectious diseases clinic ≈2 months after the onset of symptoms. Although the patient did not recall any insect bites, her adult son recalled a small punctate lesion in the central part of the initial rash. Other than the bike rides, her only risk factor for tick or mosquito exposure was working in the flower garden in her yard. She did note that there were frequently deer on the property and that the family dog often slept in her bed. She had not traveled outside the local area during the previous year. Vital signs were within reference limits, and her examination was notable only for slow responses to questions and difficulty recalling recent events. Laboratory tests for tickborne and other infectious diseases, including Lyme disease, spotted fever rickettsiosis, ehrlichiosis, and α-gal syndrome, were ordered. No antibiotics were prescribed during the visit.

Results of the Lyme disease enzyme immunoassay were positive. The sample was reflexed to a Western blot, which showed positive results (6 of 10 IgG bands reactive). The patient was prescribed a 28-day course of oral doxycycline. Substantial improvement in her mood, cognitive function, and energy levels were noted within 3 days. She completed the course of doxycycline without issue. At follow-up 1 month later, the patient reported feeling at her recent baseline, and her children no longer expressed concerns over her health. A mildly elevated α-gal result was discussed, but the patient was not experiencing any symptoms associated with the consumption of mammalian meat products.

## Conclusions

Given the relatively mild manifestations of early symptoms during Lyme disease, most patients are seen in the outpatient setting. Therefore, primary care providers play an important role in the diagnosis and management of Lyme disease and are key targets for outreach. We believe the following 2 topics merit mention. First, in 2019, the Centers for Disease Control and Prevention approved the use of a modified 2-tier test in which the traditional Western blot is replaced by a second enzyme immunoassay, which is easier to interpret and has improved sensitivity in early disease (*10*–*12*). Some commercial laboratories in North Carolina have already transitioned to the modified 2-tier test. Second, postexposure prophylaxis with a single 200-mg dose of doxycycline has not routinely been used but warrants consideration in many areas of the state if other criteria are met (*13*,*14*).

Although the patient did not have obvious exposures to ticks, her clinical manifestations were highly suggestive of Lyme disease. In addition to the nonspecific constitutional symptoms, such as malaise, she also had a large erythema migrans rash that appeared within 1 week of the likely exposure, followed by Bell’s palsy approximately 1 month later. During that period, she had visits with multiple clinicians and underwent a wide range of testing but never had specific testing or treatment for Lyme disease. Those delays, especially in the context of southward expansion of the disease along the Appalachian Mountains, highlight the need for greater awareness and professional education among healthcare providers in North Carolina (*2*,*3*).
